# Supplementary material for: Bmal1 regulates circadian expression of cytochrome P450 3a11 and drug metabolism in mice
Source: Commun Biol. 2019 Oct 16;2:378. doi: 10.1038/s42003-019-0607-z (PMC6795895; doi:10.1038/s42003-019-0607-z)
Supplement: Supplementary file 1 — Supplementary information [file 42003_2019_607_MOESM1_ESM.pdf]

Supplementary Figures

Supplementary Figure 1. (A) The map of pGL4.10 vector (promega). (B) The map of pRL-TK vector (promega).

A

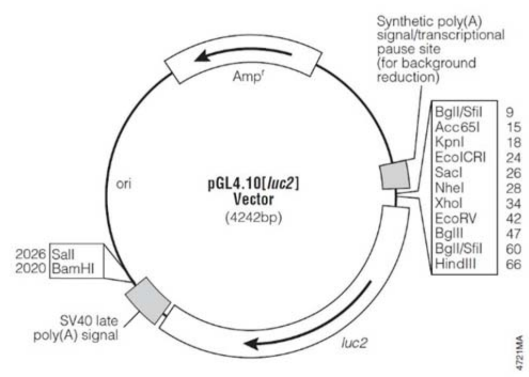

B

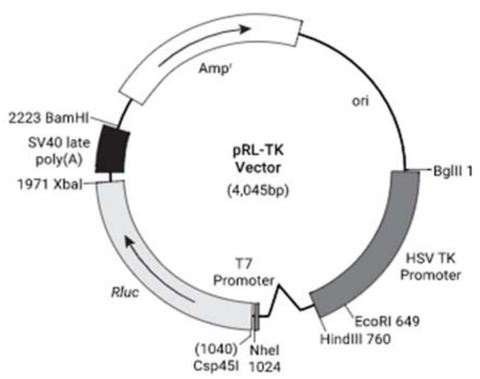

Supplementary Figure 2. The schematic diagram of a deleted DNA region of *Bmal1* gene in Bmal1-deficiency mice.

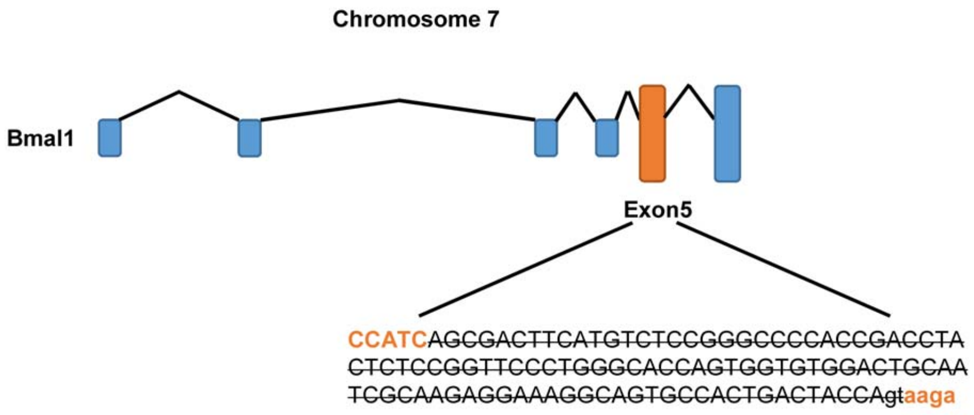

Supplementary Figure 3. Circadian Bmal1 protein expression in the livers of *WT* and *Bmal1*$^{-/-}$ mice. Data are mean ± SD (n=5). *P < 0.05 for two group comparisons at individual time points (post hoc Bonferroni test).

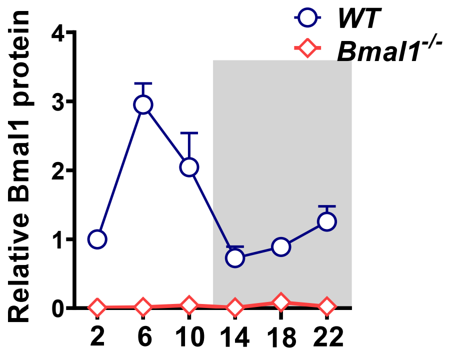

Supplementary Figure 4. (A) Circadian Cyp3a11 protein expression in the livers of *WT* and *Bmal1*$^{-/-}$ mice. **(B)** Cyp3a11 protein expression in Hepa1-6 cells transfected with Bmal1. Data are mean ± SD (n=6). *P < 0.05 (t test).

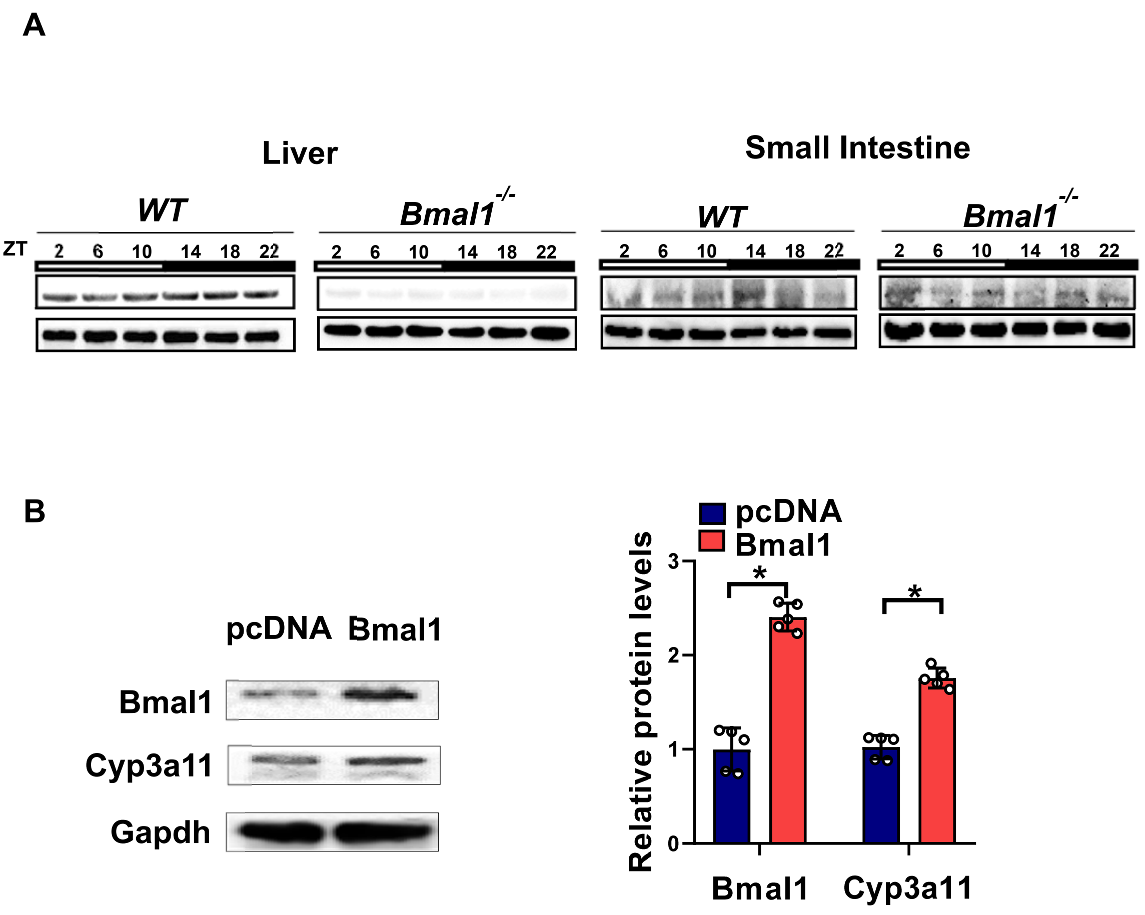

Supplementary Figure 5. (A) Circadian Dbp and Hnf4α protein expression in the livers of *WT* and *Bmal1*$^{-/-}$ mice. **(B)** Circadian Dbp and Hnf4α protein expression in the intestines of *WT* and *Bmal1*$^{-/-}$ mice.

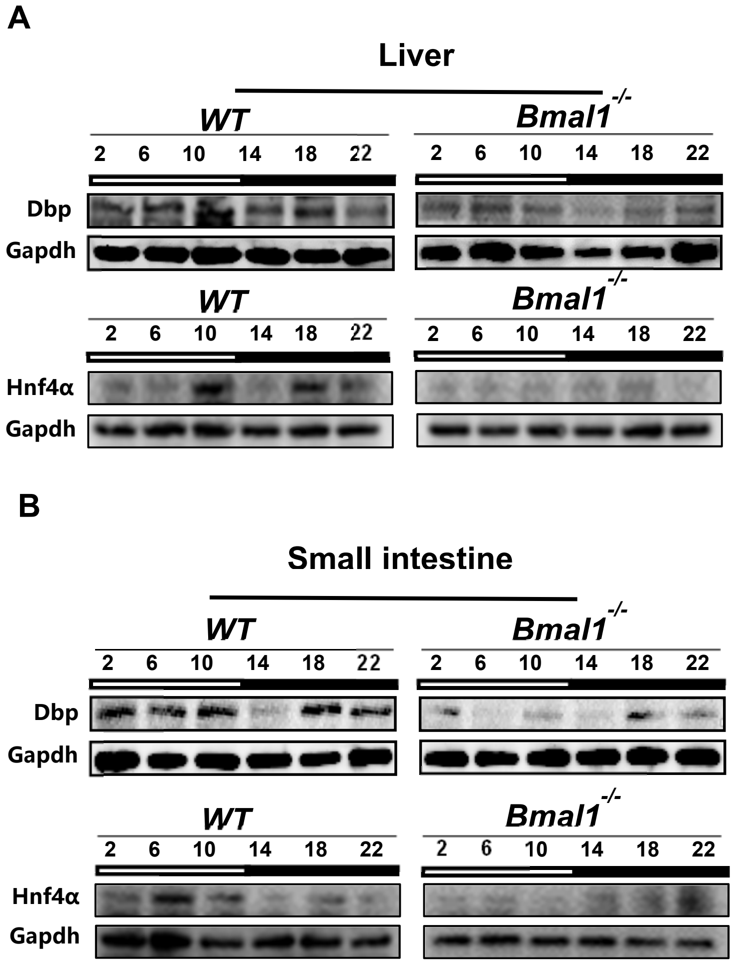

Supplementary Figure 6. Circadian Tef and Hlf mRNA expressions in livers and intestines of WT and Bmal1$^{-/-}$ mice. Data are mean ± SD (n=5). *P < 0.05 for two group comparisons at individual time points (post hoc Bonferroni test).

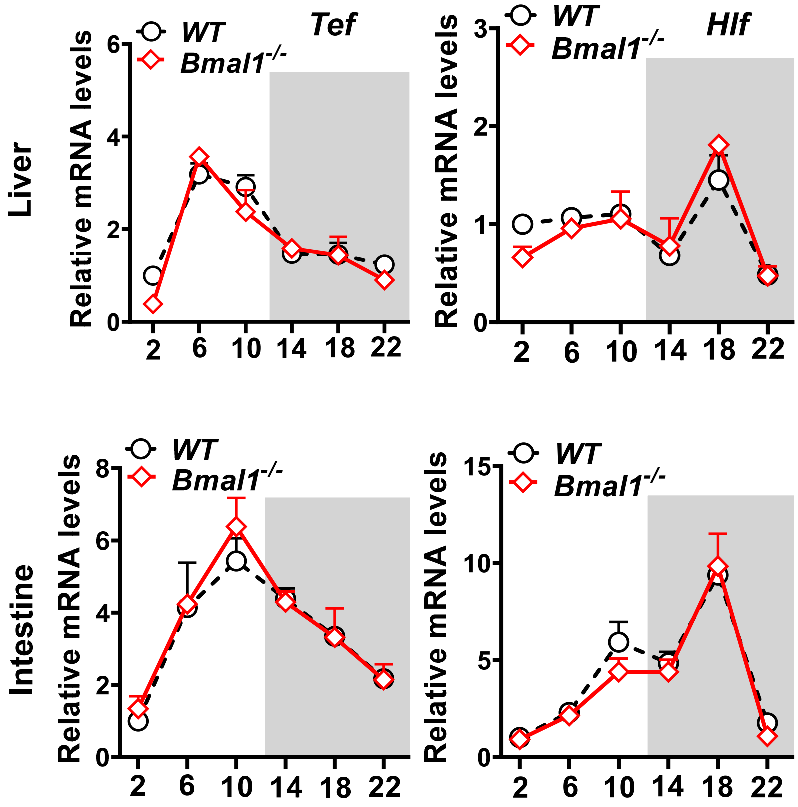

Supplementary Figure 7. Effects of Hnf4α on *Cyp3a11* reporter activity (-2.0 kb, -1.34 kb and -0.22 kb). Data are mean ± SD (n=6). *P < 0.05 (t test). n.s., not significant.

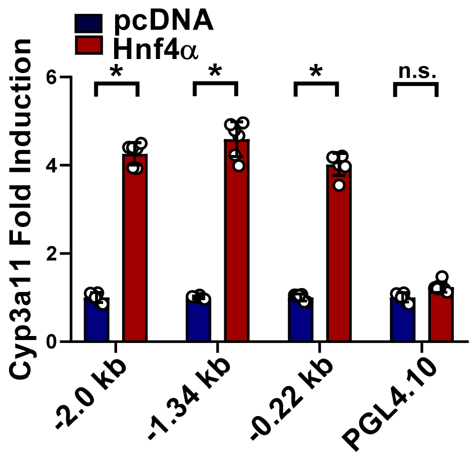

Supplementary Figure 8. (A) The effects of siDbp on the protein levels in Hepa-1c1c7 cells. **(B)** The effects of siHnf4α on the protein levels in Hepa-1c1c7 cells. Data are mean ± SD (n=5). *P < 0.05 (t test).

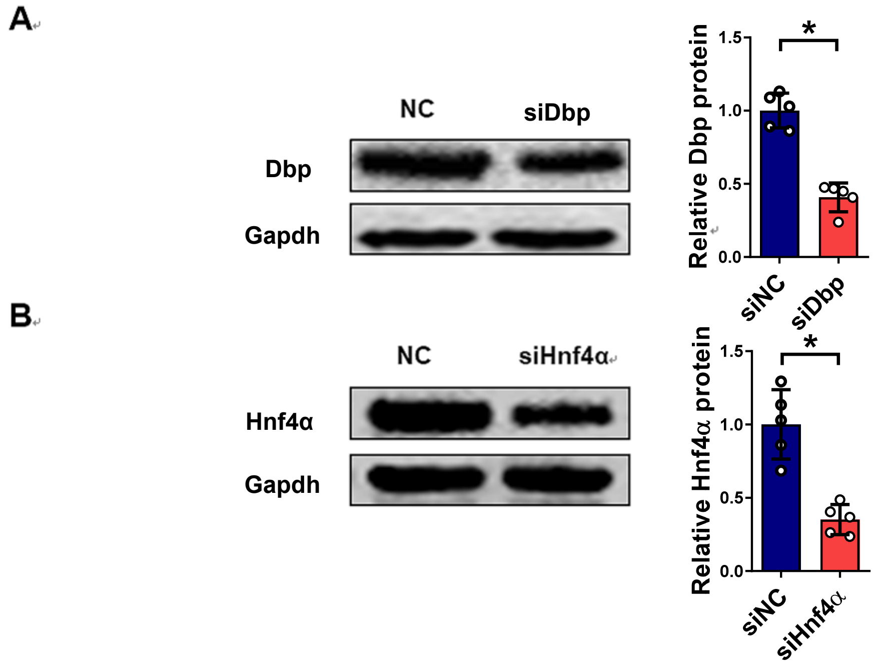

Supplementary Figure 9. The protein expression showing Bmal1 overexpression in the Hepa1-6 cells transfected the Bmal1 plasmid. Data are mean ± SD (n=5). *P < 0.05 (t test).

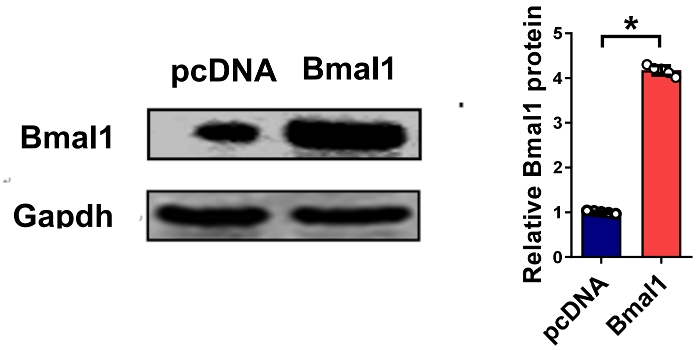

Supplementary Figure 10. (A). Four metabolites were generated from aconitine as previously reported (Tang et al., 2011). **(B)**. Activity inhibition assays, showing Cyp3a11 was the main enzyme for aconitine metabolism. Aconitine (10 μM) was incubated with mouse liver microsomes in the presence or absence of Cyp inhibitors [i.e., quercetin (Cyp1a), vitamin K3 (Cyp2c29), quinidine (Cyp2d22), 4-methylimidazole (Cyp2e1), ketoconazole (Cyp3a11)] (Hrycay and Bandiera). Data are mean ± SD (n=5). **(C)**. Two metabolites were generated from triptolide as previously reported (Du et al., 2011). **(D)**. Activity inhibition assays, showing Cyp3a11 was the main enzyme for triptolide metabolism. Triptolide (10 μM) was incubated with mouse liver microsomes in the presence or absence of Cyp inhibitors. Data are mean ± SD (n=5).

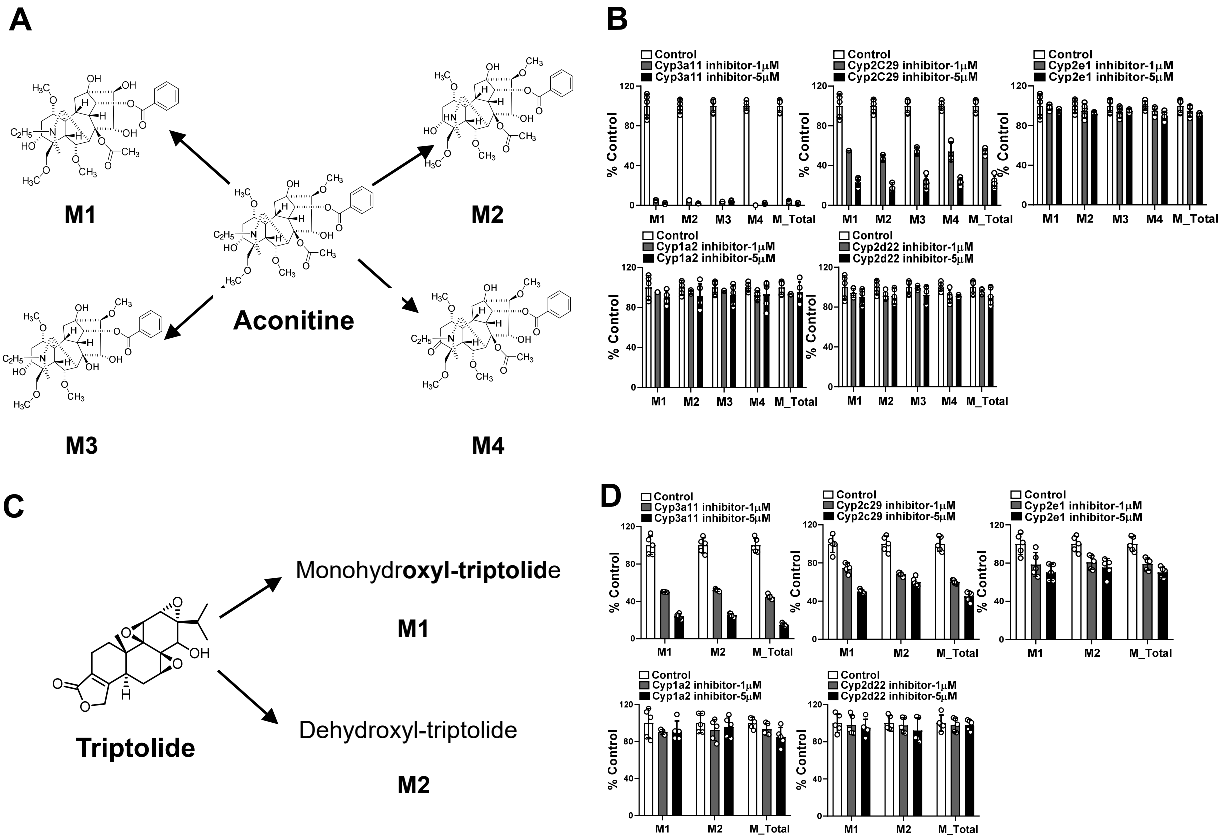

Supplementary Figure 11. Effects of Dbp and/or Hnf4α (P1 transcript) on *Cyp3a11* activity in Hepa 1-6 cells. Data are mean ± SD (n=5). *P < 0.05 (t test).

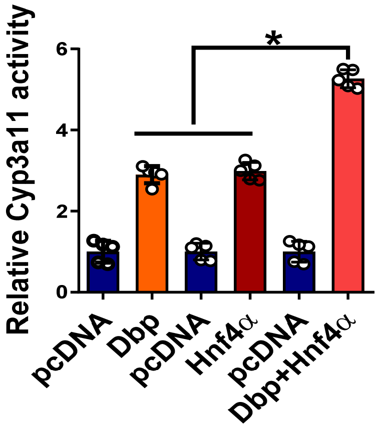

Supplementary Figure 12. Circadian Bmal2 mRNA expression in livers of WT and *Bmal1*$^{-/-}$ mice. Data are mean ± SD (n=5).

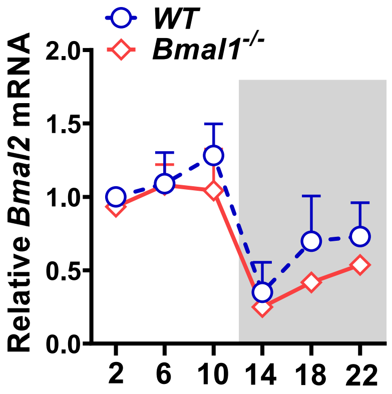

Supplementary Figure 13. Circadian Por and Alas1 mRNA expression in livers of WT and *Bmal1*$^{-/-}$ mice. Data are mean ± SD (n=5).

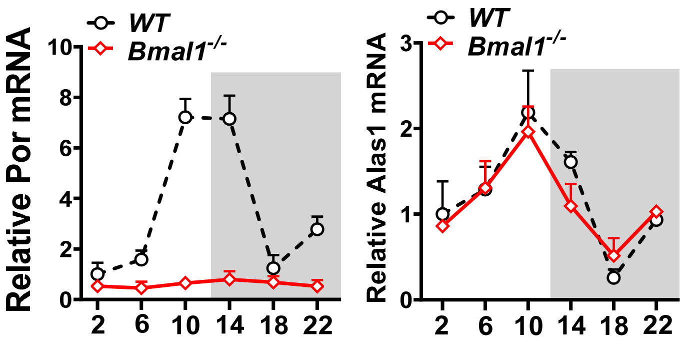

Supplementary Figure 14. Uncropped scans of representative Western blots.

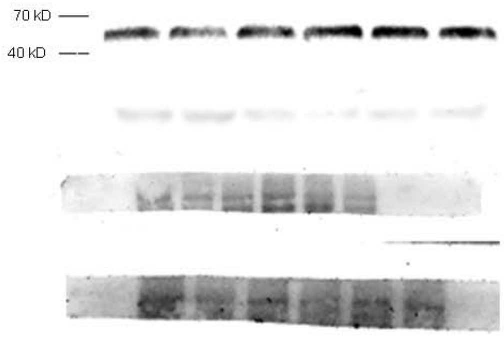

Supplementary Fig 4: Cyp3a11

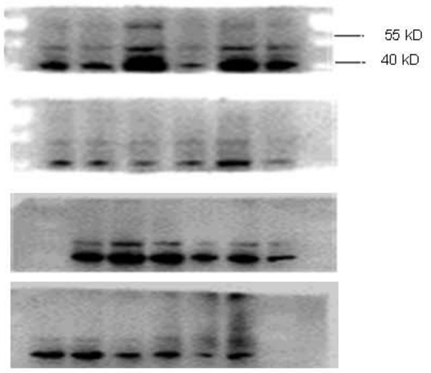

Supplementary Fig 5: Hnf4α

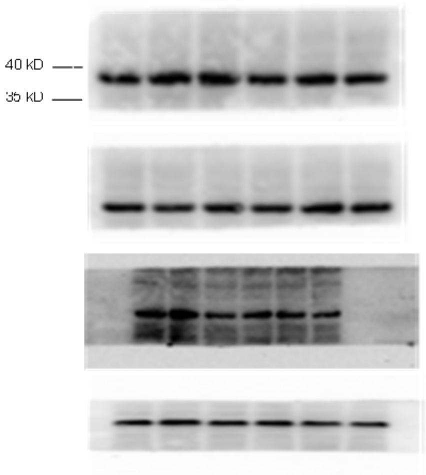

Supplementary Fig 5: Gapdh

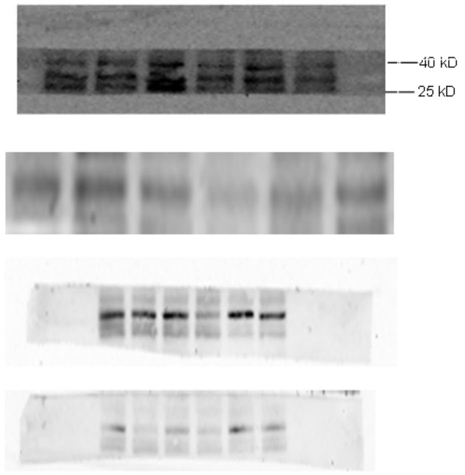

Supplementary Fig 5: Dbp

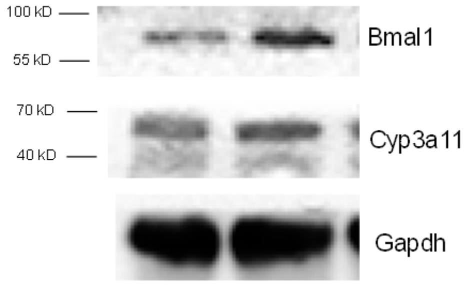

Supplementary Tables

Supplementary Table 1 Primer sequences for PCR amplification of gene promoters.

Gene promoter	Forward (5'-3' sequence)	Reverse (5'-3' sequence)
Cyp3a11 (2.0 k)	AGCTCGCTAGCCTCGAGATTAAGAGTTAGAGGACTAGGAATTCTGCTC	GCCGCCGAGGCCAGATCTCCCTGCTTGTTTGTCAGCAA
Cyp3a11 (1.34 k)	CGGTACCTGAGCTCGCTAGCCTCGAGGAGACTTTGTTGTTTTTTTCTACTTAATCT	TTGGCCGCCGAGGCCAGATCTCCCTGCTTGTTTGTCAGCA
Cyp3a11 (0.85 k)	CGGTACCTGAGCTCGCTAGCTTCCACAAGAATCCTTGGGC	TTGGCCGCCGAGGCCAGATCTCCCTGCTTGTTTGTCAGCA
Cyp3a11 (0.4 k)	CGGTACCTGAGCTCGCTAGCCTCGAGTTTTTTGAGACATGTACTCCCTTA	TTGGCCGCCGAGGCCAGATCTCCCTGCTTGTTTGTCAGCA
Cyp3a11 (0.22 k)	CGGTACCTGAGCTCGCTAGCCTCGAGACTTTTACCCATCTGGCACTGTT	TTGGCCGCCGAGGCCAGATCTCCCTGCTTGTTTGTCAGCA
Hnf4α-p1 (6.0 k)	TGAGCTCGCTAGCCTCGAGCGGATGTTCCAGCTGCCTTTA	GTACCGGATTGCCAAGCTTGGGAGACTCAGCCTGGCGT
Hnf4α-p1 (5.9 k)	CCTGAGCTCGCTAGCCTCGAGAGGGTTAGGGGTGAGACAAAT	TTTGGGTAATACATAAGATCTAAAAATAACTGTCAAGGTGCGTTG
Hnf4α-p1 (2.0 k)	GAGCTCGCTAGCCTCGAGGCACACCAGGTCTGACTTAGA	CAGTACCGGATTGCCAAGCTTGGGAGACTCAGCCTGGCGT
Hnf4α-p2 (2.0 k)	TACCTGAGCTCGCTAGCCTCGAGGGTTGAATCCTAGAGAGG	GTACCGGATTGCCAAGCTTCATGGAGGAGCAGGGGCCCA
Cyp3a11-Ebox-mut	GAGCTCGCTAGCCTCGAGGGTTGAATCCTAGAGAGGTGAGC	GTACCGGATTGCCAAGCTTCATAAGGACTCGCCACTGGA
Cyp3a11-Dbox-mut	CCGCCCATTCTCCCAAAAAAAAAATTACTGCAGGCTGTCCTCA	GACAGCCTGCAGTAATTTTTTTTTGGGAGAATGGGCGGCACCA
Cyp3a11-DR1-mut	ACTATACGAACTGCCTAAAAAAAAAAAAACCAAAGTCCAGTGATGCAA	CATCACTGGACTTTGGTTTTTTTTTTTTTAGGCAGTTCGTATAGTTCA

Supplementary Table 2 Primer sequences for qPCR assays.

m: mouse; h: human

Gene	**Forward (5'-3' sequence)**	**Reverse (5'-3' sequence)**	**Self complementarity (F/R)**	**Self 3' complementarity(F/R)**
mBmal1	CTCCAGGAGGCAAGAAGATTC	ATAGTCCAGTGGAAGGAATG	7/7	4/0
mBmal1 (knockout)	AGCGACTTCATGTCTCCG	GCTCTTACTAGTCAGTGGCA	5/8	2/3
mCar	ATGCAGGGTTCCAGTACGAG	TTGGGTAACTCCGGGTCTGT	4/4	2/0
mCyp3a11	GTGCTCCTAGCAATCAGCTT	CAGTGCCTAAAAATGGCAGAGG	5/4	3/0
mDbp	ACATCTAGGGACACACCCAGTC	AAGTCTCATGGCCTGGAATG	4/4	1/3
mFxr	GCACGCTGATCAGACAGCTA	CAGGAGGGTCTGTTGGTCTG	8/3	3/1
mHnf4α-p1	GGATATGGCCGACTACAGCG	AGATGGGGACGTGTCATTGC	4/4	2/2
mPxr	TCCCTCTTCTCCCCAGATCG	GTGAGGGCAAACCTCTCCTG	4/4	2/1
mVdr	ACGCTATGACCTGTGAAGGC	TTGAAGGGGCAGGTGAACAG	3/3	2/1
18s	CGGACAGGATTGACAGATTGATAGC	TGCCAGAGTCTCGTTCGTTATCG	3/5	2/4
mBmal1 (for PCR)	GCCCATGACCTCAGTGCCACA	CCGGCTTTAAGGCTATTGATTT	/	/
hBMAL1	ACTTCCCCTCTACCTGCTCA	ATCCAGCCCCATCTTTGTGG	2/3	2/2
hCYP3A4	GTGGGGCTTTTATGATGGTCA	ACATCTCCATACTGGGCAATGA	3/4	3/1
GAPDH	CATGAGAAGTATGACAACAGCCT	AGTCCTTCCACGATACCAAAGT	4/3	3/1

Supplementary Table 3. Incubation conditions for Cyp3a11 activity assays.

Regent	Volume (μl)
KPI buffer	191
100 mM NADPH	2
50 mM $MgCl_2$	2
Microsomes (final concentration is 1 mg/ml)	2
Substrates (final concentration is 10 μM)	2

Supplementary Table 4. Primer sequences for ChIP assays.

Gene	Forward (5'-3' sequence)	Reverse (5'-3' sequence)	Self complementarity (F/R)	Self 3' complementarity(F/R)
Hnf4α-p1-promoter	GTGCCTGCCTTGGAAGATTG	GGTTAATCACCCCCGTTGCT	4/5	0/1
Hnf4α-p1-enhancer	ACAAGCCCCTGCTTCACATC	TCCTGGGTTATGCAAGAGGC	4/4	0/2
Dbp	TGGGACGCCTGGGTACAC	GGGAATGTGCAGCACTGGTT	4/7	4/1

Supplementary Table 5. Oligonucleotide sequences for EMSA assays.

Oligonucleotide	Forward (5'-3' sequence)	Reverse (5'-3' sequence)
Hnf4α-Ebox-1	TTCGGATGTTCCAGCTGCCTTTATCTCC	GGAGATAAAGGCAGCTGGAACATCCGAA
Hnf4α-Ebox-1-mut	TTCGGATGTTCATCGATCCTTTATCTCC	GGAGATAAAGGATCGATGAACATCCGAA
Hnf4α-Ebox-2	TGGAGCAGGGCACATGCAGGCTGTTGAC	GTCAACAGCCTGCATGTGCCCTGCTCCA
Hnf4α-Ebox-2-mut	TGGAGCAGGGATATGCCAGGCTGTTGAC	GTCAACAGCCTGGCATATCCCTGCTCCA
Hnf4α-Ebox-3	AGGCTGCTGGACATGTGTGGAGAGGCCA	TGGCCTCTCCACACATGTCCAGCAGCCT
Hnf4α-Ebox-3-mut	AGGCTGCTGGAATGCGCTGGAGAGGCCA	TGGCCTCTCCAGCGCATTCCAGCAGCCT

Supplementary Table 6. Cosinor analyses for mRNA of transcription factors. Original point was set at ZT17 (=12 PM). Data are recorded as mean ± SE (standard error).

Gene			Acrophase (h)	Mesor (h)	Amplitude	p values
WT	Cyp3a11	Liver	13.44±0.01	2.09±0.02	1.08±0.01	0.019
		Intestine	12.75±0.09	1.74±0.10	0.53±0.02	0.022
	Hnf4α	Liver	21.04±0.10	1.10±0.09	1.19±0.01	0.023
		Intestine	20.24±0.54	1.07±0.03	0.33±0.02	0.018
	Dbp	Liver	14.14±0.43	1.59±0.01	1.41±0.04	0.031
		Intestine	14.93±0.23	2.18±0.10	3.09±0.07	0.034
	Pxr	Liver	21.29±0.67	1.50±0.23	0.41±0.05	0.027
		Intestine	15.56±0.24	0.97±0.11	0.23±0.07	0.034
	Car	Liver	12.36±0.23	0.95±0.12	0.21±0.04	0.029
		Intestine	11.04±0.31	0.63±0.03	0.32±0.02	0.020
	Vdr	Liver	9.05±0.20	0.66±0.09	0.28±0.03	0.049
		Intestine	8.43±0.19	0.75±0.08	0.22±0.07	0.040
	Fxr	Liver	14.67±0.59	1.21±0.09	0.37±0.04	0.040
		Intestine	7.44±0.09	0.79±0.02	0.15±0.02	0.059
KO	Cyp3a11	Liver	13.85±0.45	0.88±0.02	0.16±0.01	0.102
		Intestine	12.72±0.21	1.16±0.04	0.12±0.01	0.267
	Hnf4α	Liver	19.22±0.23	0.68±0.02	0.13±0.02	0.087
		Intestine	18.78±0.90	0.57±0.03	0.17±0.02	0.102
	Dbp	Liver	13.18±0.79	0.45±0.04	0.74±0.02	0.045
		Intestine	13.17±0.20	0.49±0.04	0.76±0.05	0.053
	Pxr	Liver	20.58±0.93	1.56±0.09	0.48±0.04	0.032
		Intestine	15.62±1.04	1.03±0.03	0.29±0.02	0.039
	Car	Liver	13.04±0.81	0.89±0.09	0.23±0.02	0.036
		Intestine	11.79±0.03	0.73±0.03	0.30±0.04	0.012
	Vdr	Liver	9.09±0.10	0.71±0.02	0.27±0.01	0.069
		Intestine	10.61±0.21	0.82±0.10	0.19±0.01	0.093
	Fxr	Liver	14.55±0.04	1.15±0.13	0.25±0.01	0.045
		Intestine	8.44±0.40	0.93±0.05	0.19±0.01	0.076

Supplementary Table 7. Cosinor analyses for protein of Cyp3a11, Hnf4α and Dbp. Original point was set at ZT17 (=12 PM). Data are recorded as mean ± SE (standard error).

Gene			Acrophase(h)	Mesor (h)	Amplitude	p values
WT	Cyp3a11	Liver	1.64±0.02	1.75±0.09	1.05±0.09	0.013
		Intestine	23.08±0.92	1.12±0.10	0.17±0.01	0.011
	Hnf4α	Liver	17.44±0.39	1.04±0.02	0.35±0.03	0.029
		Intestine	12.48±0.89	0.75±0.13	0.38±0.04	0.034
	Dbp	Liver	16.48±0.04	1.23±0.19	0.63±0.05	0.013
		Intestine	12.28±0.10	0.97±0.15	0.31±0.04	0.022
KO	Cyp3a11	Liver	8.96±0.39	0.59±0.10	0.15±0.01	0.369
		Intestine	0.19±0.04	0.81±0.11	0.13±0.03	0.556
	Hnf4α	Liver	20.69±1.93	0.82±0.10	0.19±0.09	0.866
		Intestine	10.98±0.76	0.56±0.10	0.14±0.04	0.259
	Dbp	Liver	16.43±0.45	0.71±0.12	0.32±0.01	0.128
		Intestine	10.12±0.48	0.41±0.06	0.12±0.02	0.033

Supplementary References

1 Du F, Liu T, Liu T, Wang Y, Wan Y, Xing J. Metabolite identification of triptolide by data-dependent accurate mass spectrometric analysis in combination with online hydrogen/deuterium exchange and multiple data-mining techniques. Rapid Commun Mass Spectrom. 2011;25(20):3167-77.

2 Hrycay EG, Bandiera SM. Expression, function and regulation of mouse cytochrome P450 enzymes: comparison with human P450 enzymes. Curr Drug Metab. 2009;10(10):1151-83.

3 Tang L, Ye L, Lv C, Zheng Z, Gong Y, Liu Z. Involvement of CYP3A4/5 and CYP2D6 in the metabolism of aconitine using human liver microsomes and recombinant CYP450 enzymes. Toxicol Lett. 2011;202(1):47-54.
