# Supplementary material for: Bmal1 regulates circadian expression of cytochrome P450 3a11 and drug metabolism in mice
Source: Commun Biol. 2019 Oct 16;2:378. doi: 10.1038/s42003-019-0607-z (PMC6795895; doi:10.1038/s42003-019-0607-z)
Supplement: Supplementary file 2 — Description of additional supplementary items [file 42003_2019_607_MOESM2_ESM.docx]

**Description of Additional Supplementary Files**

**File Name**: Supplementary Data 1

**Description**:  Excel file with source data used for main figures.
